# Solvent‐Tuned DLP 3D‐Printed PNVCL Hydrogels With Enhanced Thermoresponse

**DOI:** 10.1002/marc.70429

**Published:** 2026-09-16

**Authors:** Charlotte Dai, Frédéric Demoly, Vincent Lapinte, Sébastien Blanquer

**Affiliations:** ^1^ ICGM CNRS ENSCM University of Montpellier Montpellier France; ^2^ CNRS UTBM ICB UMR 6303 Université Marie et Louis Pasteur Belfort Cedex France; ^3^ Institut Universitaire De France (IUF) Paris France

**Keywords:** 3D/4D printing, digital light processing, poly(N‐vinylcaprolactam), porous hydrogel, response kinetics, smart hydrogel

## Abstract

The development of Poly(N‐vinylcaprolactam) (PNVCL)‐based hydrogels for additive manufacturing remains challenging due to the difficulty of simultaneously achieving printability and enhanced thermoresponsive performance. Herein, chemically crosslinked P(NVCL‐DVA) organohydrogels (DVA, 3,9‐divinyl‐2,4,8,10‐tetraoxaspiro[5.5]undecane) were prepared using diethylene glycol (DEG) as a cosolvent with water and fabricated by digital light processing (DLP) 3D printing, followed by solvent exchange with water. The influence of formulation parameters, particularly DEG content, on network formation and thermoresponsive behavior was systematically investigated. The optimized hydrogels exhibited interconnected porous structures associated with high water uptake, enhanced deswelling amplitude, and rapid deswelling and reswelling kinetics. Collectively, these findings establish an effective formulation strategy that expands the potential of PNVCL‐based materials for DLP 3D printing.

## Graphical abstract

DLP 3D printing of PNVCL hydrogels remains challenging due to a trade‐off between printability and thermoresponsive performance. Here, DEG‐tuned P(NVCL‐DVA) organohydrogels were successfully 3D‐printed and subsequently solvent‐exchanged with water. The optimized hydrogels exhibited interconnected porous structures, high water uptake and rapid thermo‐responsive kinetics.

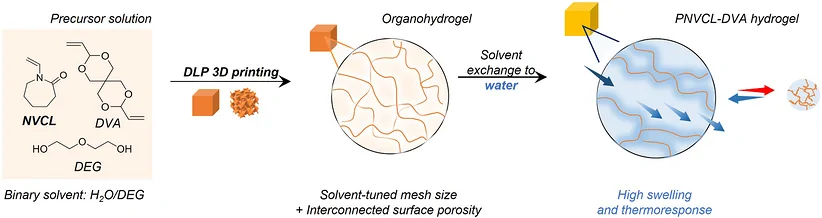

## 1 Introduction

Additive manufacturing of thermoresponsive hydrogels offers potential for applications in soft robotics [1], drug delivery [2], and tissue engineering [3], owing to the design freedom enabled by layer‐by‐layer fabrication of complex 3D structures. Chemically crosslinked hydrogels based on lower critical solution temperature (LCST) polymers are particularly attractive because they can undergo macroscopic volume change upon heating above their LCST. Poly(N‐isopropylacrylamide) (PNIPAM) remains the most extensively studied LCST polymer due to its transition temperature around 32°C, close to physiological conditions, and its large and reversible volume change [4, 5, 6]. However, concerns regarding the toxicity of the degradation products of PNIPAM have motivated the exploration of alternative thermoresponsive polymers [7]. In particular, Poly(N‐vinylcaprolactam) (PNVCL) has emerged as a promising candidate due to its LCST behavior near physiological temperature around 32°C, depending on the molecular weight, and its favorable biocompatibility compared with PNIPAM [8, 9]. Nevertheless, the macroscopic shrinkable behavior of chemically crosslinked PNVCL hydrogels remains comparatively less explored. In addition, research on 3D‐printed PNVCL hydrogels is still at an early stage, with efforts mainly focused on photocurable resins containing the monomeric derivative N‐vinylcaprolactam (NVCL).

Geever et al. [10, 11] first demonstrated stereolithography (SLA) printing of cylinder, jigsaw, and flower‐shaped PNVCL hydrogels, whereas more complex geometries have been obtained by casting precursor solutions into 3D‐printed molds. García‐Sobrino et al. [12] used extrusion‐based printing to create polyvinyl alcohol (PVA) sacrificial molds, facilitating the casting of non‐planar PNVCL‐based hydrogels for tissue engineering applications. Direct 3D printing of complex PNVCL hydrogel architectures thus remains limited, which could be explained by the low reactivity of the NVCL monomer compared to other vinyl monomers [8], combined with its limited water solubility. To address these constraints, previous studies have employed aqueous emulsions to maintain NVCL at soluble concentrations [13, 14], ethanol‐based solvents [12, 15, 16, 17], or NVCL heated above its melting point [10, 12, 18, 19, 20, 21]. However, these approaches may compromise thermoresponsive properties due to insufficient or excessive PNVCL content. Volatile solvents can cause changes in viscosity during processing and introduce evaporation‐induced defects, compromising print fidelity and repeatability. Processing molten NVCL requires preheating, which introduces the risk of recrystallization or the need to maintain elevated temperatures during printing, thereby restricting compatibility with certain 3D printing techniques. Beyond processing challenges, PNVCL hydrogels typically exhibit moderate shrinkage amplitudes and relatively slow swelling and deswelling kinetics [10, 11, 12, 13, 14, 15, 16, 17, 18, 19, 20, 21]. Therefore, identifying a compatible solvent that ensures NVCL solubility while preserving thermoresponsive behavior and printability is critical.

Several studies have highlighted how solvent‐polymer interactions during network formation can influence porosity and water transport. Specifically, varying solvent compositions has proven to be effective in preparing high‐performance thermoresponsive PNIPAM‐based systems. Alsaid et al. [22] reported that PNIPAM hydrogels prepared using a dimethyl sulfoxide (DMSO)‐water co‐nonsolvent mixture exhibited open and interconnected porous structures, which resulted in enhanced water permeability. Cho et al. [23] used a carbonyl‐affine solvent to extend polymer chain length between entanglements, which enabled the preparation of mechanically robust PNIPAM hydrogels with improved shrinkage actuation. In addition, Liu et al. [24] combined polymerization‐induced phase separation (PIPS) in ethylene glycol (EG) with 3D printing to fabricate PNIPAM hydrogels bearing hierarchical porosity, both sub‐micrometer and millimeter‐sized pores, which enabled rapid deswelling and reswelling.

Herein, we aim to enhance the thermoresponsive properties of 3D‐printed PNVCL hydrogels by preparing photocrosslinked organohydrogels using a mixture of water‐diethylene glycol (H_2_O/DEG) before solvent exchange to water. DEG was selected as a water‐miscible cosolvent compatible with digital light processing (DLP) 3D printing. Precursor organohydrogel formulations were first optimized to yield self‐standing hydrogels suitable for DLP 3D printing, before comparing the properties of hydrogels prepared with increasing DEG in the precursor solutions. Increasing DEG content resulted in lower gel fraction, larger mesh size, and higher swelling difference between temperatures below and above the LCST of PNVCL. The enhanced deswelling amplitude was achieved without compromising deswelling and reswelling kinetics. To demonstrate the applicability of this strategy to complex architectures, gyroid structures were fabricated by DLP 3D printing. Overall, these findings highlight that solvent composition provides an effective strategy for tuning the thermoresponsive behavior of 3D‐printable PNVCL‐based hydrogels.

## 2 Results and Discussion

### 2.1 Preparation of Self‐Standing PNVCL Hydrogels

Chemically crosslinked PNVCL organohydrogels were prepared via free radical photopolymerization in a binary water/organic solvent system (Figure 1). The low solubility of the NVCL monomer in water required the use of a cosolvent to dissolve the monomer at higher concentrations. NVCL was soluble in ethanol, dimethyl sulfoxide, and DEG at molal concentrations up to the maximum tested concentration of 8 mol kg^−1^ (Figure S1A). DEG was, however, preferred as a cosolvent because it was miscible with water and more suitable for DLP 3D printing compared to more volatile solvents. The solvent composition ratio H_2_O/DEG was first set to 50/50 v/v, as this amount of DEG was sufficient to fully solubilize NVCL monomer on a 1‐g scale. Solvent exchange to water was subsequently performed to remove DEG from organohydrogels, which was verified by ^1^H NMR analysis of the washing bath used during the exchange process (Figure S1B, peaks at 3.5–3.8 ppm). TGA analysis of washed and dried samples also showed limited traces of solvent with a mass loss around 100°C attributable to residual water (Figure S1C).

**FIGURE 1:**
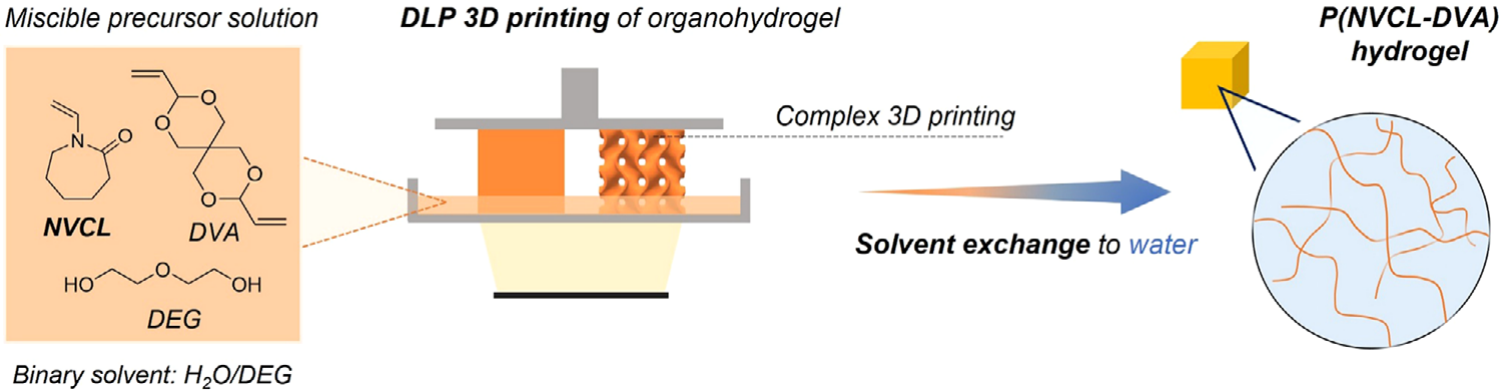
Strategy for DLP 3D‐Printable PNVCL‐based hydrogels. Schematic of the preparation steps with components of the precursor organohydrogel solution (see Experimental Section).

Crosslinker type was found to be a key determinant in the preparation of self‐standing hydrogels with high gel fraction and good manipulability (Figure 2; Table 1). The effect of crosslinker type was investigated by assessing properties of hydrogels prepared using crosslinker polyethylene glycol diacrylate (PEGDA) of different chain lengths (PEGDA_250_ or PEGDA_700_) or 3,9‐divinyl‐2,4,8,10‐tetra‐oxaspiro[5.5]undecane (DVA). PEGDA is commonly used in the literature as a photocrosslinker owing to its high reactivity under UV irradiation, while DVA was selected as a vinyl‐bearing crosslinker with lower reactivity [25] and higher solubility in DEG compared to water. Hereafter, PNVCL hydrogels prepared with different crosslinker types are referred to as Gel‐crosslinker type or Gel‐crosslinker type‐crosslinker concentration if the concentration is specified (e.g., Gel‐PEGDA_700_ or Gel‐PEGDA_700_‐2 respectively). Overall, Gel‐PEGDA had low gel fractions even at high crosslinker concentration, with values below 40% for both Gel‐PEGDA_250_‐6 and Gel‐PEGDA_700_‐6, whereas Gel‐DVA exhibited gel fractions above 70% at a crosslinker concentration of 2 mol% (Figure 2A). ^1^H NMR analysis of the soluble fraction (Figure S2A) revealed residual monomer (vinyl protons at 5–6.5 ppm) and broad signals corresponding to free PNVCL chains (methylene protons adjacent to the carbonyl group at 2.2–2.6 ppm), which confirmed the photopolymerization of the NVCL monomer, while indicating limited reaction between NVCL and PEGDA_700_. The difference in gel fractions could be explained by kinetic limitations during network formation arising from differences in reactivities between the crosslinker and the NVCL monomer. Gel point, defined as the time at which storage (*G*′) and loss modulus (*G*″) intersect, was thus assessed using in situ photorheology measurements on organohydrogel precursor solutions (Figure 2B, see Experimental Section). Gel point was approximately 8‐fold slower when DVA was used instead of PEGDA_700_ (56 s and 7 s, respectively). These findings agreed with previous studies that reported rapid radical generation and propagation could drive early gelation at relatively low monomer conversion, restricting the mobility of unreacted functional groups [26, 27].

**FIGURE 2:**
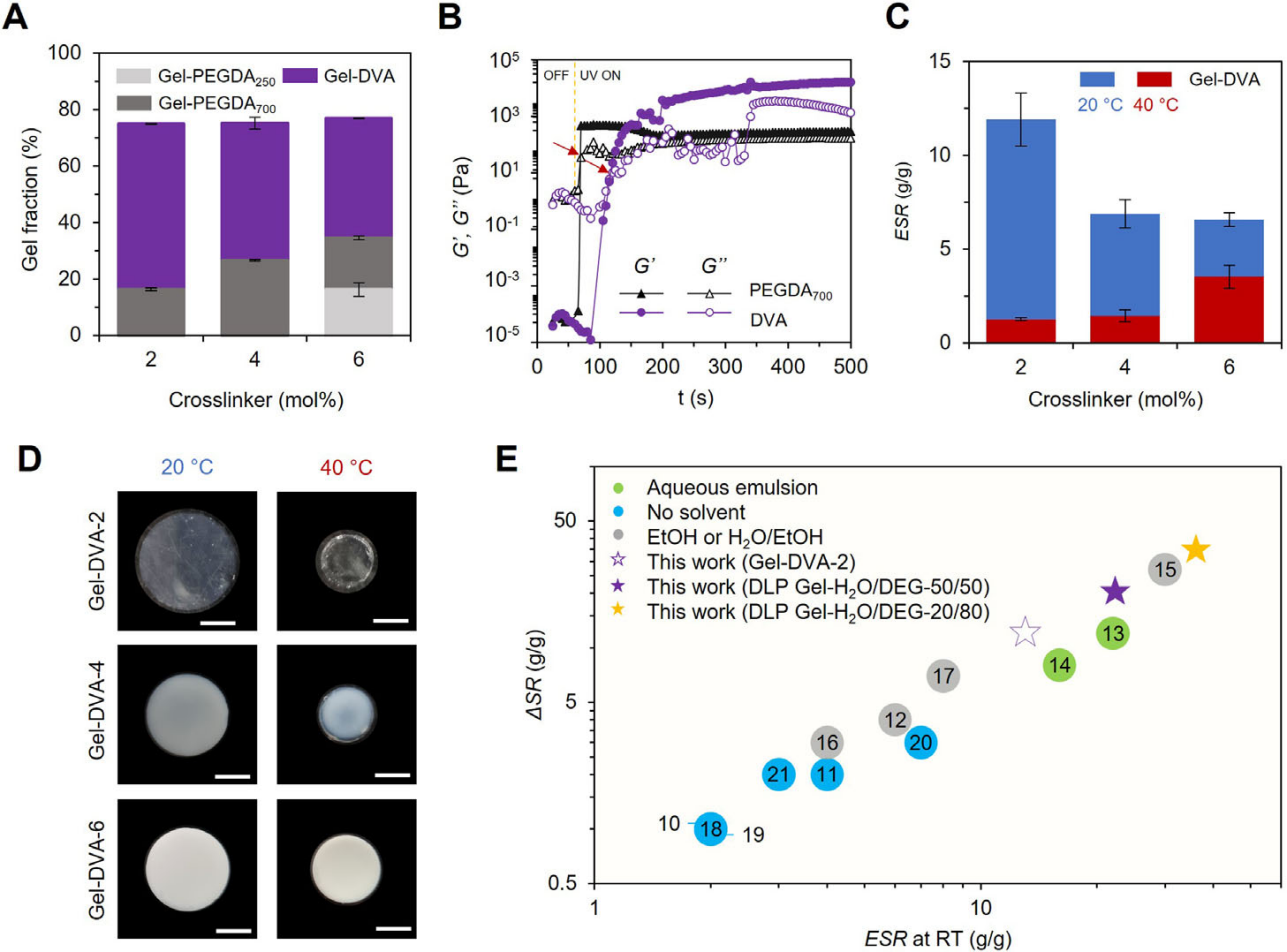
Effect of crosslinker type and concentration on the properties of PNVCL hydrogels. (A) Gel fraction suggesting better crosslinking efficiency with DVA compared with PEGDA‐based crosslinkers. (B) Photorheology measurements comparing crosslinker type, indicating early gelation with PEGDA_700_. Influence of DVA concentration on (C) *ESR_20_* and *ESR_40_*, and (D) corresponding gel diameter changes (scale bar 0.5 cm). (E) Comparison of ESR and swelling ratio differences with the literature [10, 11, 12, 13, 14, 15, 16, 17, 18, 19, 20, 21]. This chart can be considered together with Table S2 in Supporting Information for additional details on sample characteristics and testing conditions.

**TABLE 1:** Summary of tested conditions.

n°	Name	NVCL (w/v%)	Crosslinker type	Crosslinker (mol%)	LAP (w/w%)	H_2_O/DEG (v/v)
1	PEGDA_250_‐2	25	PEGDA_250_	2	2	50/50
2	PEGDA_250_‐4	25	PEGDA_250_	4	2	50/50
3	Gel‐PEGDA_250_‐6	25	PEGDA_250_	6	2	50/50
4	Gel‐PEGDA_700_‐2	25	PEGDA_700_	2	2	50/50
5	Gel‐PEGDA_700_‐4	25	PEGDA_700_	4	2	50/50
6	Gel‐PEGDA_700_‐6	25	PEGDA_700_	6	2	50/50
7^a^	Gel‐DVA‐2	25	DVA	2	2	50/50
8	Gel‐DVA‐4	25	DVA	4	2	50/50
9	Gel‐DVA‐6	25	DVA	6	2	50/50
10	Gel‐NVCL‐50	50	DVA	2	2	50/50
11	Gel‐NVCL‐75	75	DVA	2	2	50/50
12	Gel‐LAP‐0.5	25	DVA	2	0.5	50/50
13	Gel‐LAP‐4	25	DVA	2	4	50/50
14	Gel‐H_2_O/DEG‐40/60	25	DVA	2	2	40/60
15	Gel‐H_2_O/DEG‐30/70	25	DVA	2	2	30/70
16	Gel‐H_2_O/DEG‐20/80	25	DVA	2	2	20/80

^a^ Condition number 7 also corresponds to Gel‐NVCL‐25, Gel‐LAP‐2, and Gel‐H_2_O/DEG‐50/50.

It is worth noting that increasing the length of the PEGDA crosslinker allowed us to reduce the crosslinker concentration needed to form self‐standing hydrogels (2 mol% for Gel‐PEGDA_700_ and 6 mol% for Gel‐PEGDA_250_). Gel‐PEGDA_700_ exhibited good manipulability even at 2 mol% crosslinker concentration despite its low gel fraction, whereas Gel‐PEGDA_250_ samples were easily deformed or fragmented in water. These results could be explained by the increased chain length of PEGDA_700_, which could promote network formation by increasing the probability of radical crosslinking events. A similar phenomenon was previously reported for modified PEG crosslinkers based on PEG chains with molecular weights of 200, 1500, and 3400 g mol^−1^ [28]. The authors suggested that higher physical entanglements in networks prepared with higher molecular weight PEG facilitated the formation of stable gels.

DVA was therefore selected as a crosslinker for the rest of the study. Influence of DVA concentration on thermoresponsive properties was then assessed by determining the equilibrium mass swelling ratio at 20°C (*ESR_20_*) and 40°C (*ESR_40_*), below and above the theoretical volume phase transition temperature (VPTT) of PNVCL, respectively (Figure 2C), and by observing sample diameter changes between these two temperatures (Figure 2D). Since *ESR* is inversely proportional to dry mass, lower gel fraction can increase *ESR* values, complicating direct comparisons between samples with different polymer content. In contrast, diameter changes directly reflect temperature‐induced network deformation and therefore provide a more direct indicator of thermoresponsive behavior than *ESR*, especially for systems described by low gel fractions. *ESR* remains useful as it enables comparison with existing literature where it is widely reported, offers information on water absorption capacities relevant for applications such as drug delivery, and *ESR* determination offers better sensitivity for quantifying small differences between samples. For example, *ESR_20_* was highest for Gel‐PEGDA_700_‐2, which can be attributed to a corresponding low gel fraction of 14%, while Gel‐DVA‐2 exhibited the second highest *ESR_20_* due to a higher amount of NVCL monomer incorporated into the network despite its higher gel fraction (Figure S2B,C). Increasing DVA crosslinker concentration reduced *ESR_20_* and increased *ESR_40_*, consistent with more highly crosslinked structures exhibiting reduced swelling and deswelling capacities due to restricted chain mobility [29].

Interestingly, Gel‐DVA‐2 exhibited both high swelling and a large swelling difference between 20°C and 40°C, comparing favorably with previously reported PNVCL‐based hydrogels (Figure 2E and Table S2) [10, 11, 12, 13, 14, 15, 16, 17, 18, 19, 20, 21]. In particular, Gel‐DVA‐2 presented a swelling difference about 6‐times greater than that reported for photocrosslinked SLA 3D‐printed PNVCL hydrogels [10, 11]. The enhanced swelling and thermoresponse observed herein could partly be attributed to the low monomer concentration of 25 w/v%. Previous studies have reported the use of PEGDA and DVA crosslinkers at similar concentrations to this work, with high monomer concentration [10, 11, 16], which can limit hydrogel swelling by increasing network density and chain entanglements [30]. Consistently, monomer concentration had a greater effect on *ESR_20_* than crosslinker concentration (Figure S3), whereas its smaller effect on thermoresponsive behavior could be attributed to the higher PNVCL chain density at increased monomer concentrations, enhancing the capacity of the hydrogel to undergo a volume phase transition.

### 2.2 Solvent‐Tuned Properties of PNVCL Hydrogels

The effect of solvent on polymerization was first assessed by monitoring the absorbance of the photoinitiator in varying H_2_O/DEG composition ratios (Figure S4A). Increasing DEG content in the solvent reduced the maximum absorbance and induced a red shift in the wavelength of maximum absorbance, which suggested photoinitiator aggregation with increasing DEG. Although absorbance measurements only provide information on the capacity of the photoinitiator to capture light, insufficient absorbance can diminish the overall efficacy of the photoinitiator. Photorheology measurements on precursor organohydrogel solutions also indicated that DEG had an influence on polymerization and crosslinking kinetics (Figure 3A,B, see Experimental Section and Table 1, fixed parameters were NVCL 25 w/v%, DVA 2 mol%, LAP 1 w/w%). The precursor solution with an H_2_O/DEG ratio of 50/50 reached the gel point after 56 s, while increasing DEG content progressively delayed gelation, with gel points of 61 s, 68 s, and 76 s observed for H_2_O/DEG ratios of 40/60, 30/70, and 20/80, respectively. The plateau modulus from the photorheology curves suggested lower crosslinking efficiency with higher DEG content, which aligned with the lower gel fractions observed (Figure 3C, gels named Gel‐H_2_O/DEG‐solvent composition hereafter). The slower reactivity and lower network formation efficiency could be explained by the effects of DEG on photoinitiator performance as well as monomer dilution due to an increase in precursor solution viscosity with higher DEG content. An increase in DEG content could also increase termination events during polymerization, which would lead to the formation of networks with lower crosslinking density and with dangling chains [31]. Consistently, increasing photoinitiator concentration, which can promote higher termination rate [32], was found to increase gel time and decrease gel fraction with a similar variation to increasing DEG content (Figure S4B, Table S1).

**FIGURE 3:**
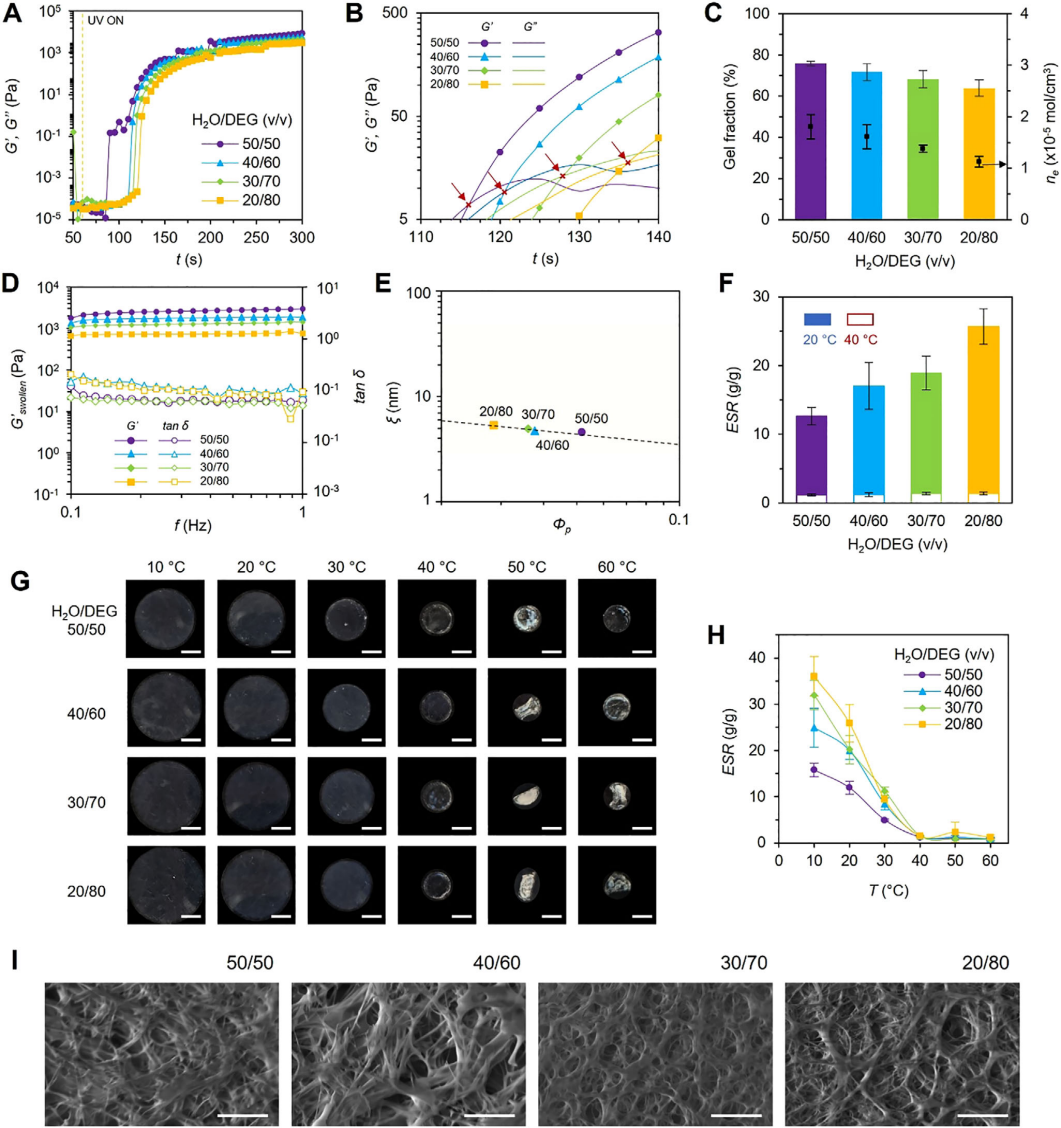
Effects of solvent composition on hydrogel properties. (A) Representative storage modulus curves showing differences in reactivities; only storage modulus is represented for clarity. (B) Zoom‐in with both storage modulus and loss modulus curves showing gel point increase with higher DEG content. (C) Gel fraction and associated crosslinking density. (D) Storage modulus of equilibrium swollen gels at 20°C. (E) Mesh size and polymer fraction, where the highlighted zone corresponds to mesoporous hydrogels (see Experimental Section). (F) *ESR_20_* and *ESR_40_*. (G) Sample diameter change with temperature (scale bar 0.5 cm), and (H) corresponding *ESR* evolution with temperature. (I) LVSEM imaging (left to right, scale bar 5 µm).

To better understand the influence of DEG on network formation, structural parameters of the polymer network were determined from rheology measurements on equilibrium swollen gels (Figure 3C–E, see Experimental Section). Mesh size was systematically calculated to determine whether shear modulus, corresponding to *G*′ values in the linear viscoelastic regime, required correction to account for water uptake by pores [33]. Mesh sizes were characteristic of mesoporous hydrogels with values between 3 and 50 nm, therefore, corrections using isostrain mixing rules were applied for further analysis of the network properties (see Experimental Section). Consistent with the lower gel fraction at higher DEG content, Gel‐H_2_O/DEG‐20/80 exhibited lower crosslinking density, increased mesh size, and increased *ESR_20_* (Figure 3C,E,F). Interestingly, *ESR_40_* was not affected by DEG content, which could be attributed to the overall high capacity of these hydrogels to deswell upon temperature increase. Sample diameters at 40°C did, however, show that Gel‐H_2_O/DEG‐20/80 was smaller than hydrogels prepared at other composition ratios (Figure 3G). Since *ESR_40_* was not affected by DEG content, Gel‐H_2_O/DEG‐20/80 exhibited both higher swelling and larger deswelling amplitude. These results indicated that variations in DEG content could enable the preparation of hydrogels with similar deswollen dimensions at 40°C to exhibit distinct swelling behaviors at 20°C, providing a straightforward way to tune properties and broaden the applicability of these hydrogels.

The volume transition occurred between 20°C and 40°C for all samples, with the most pronounced decrease in swelling observed within this temperature range (Figure 3G–H). VPTT values measured by DSC decreased with increasing DEG content (Figure S5), agreeing with the increase in VPTT observed for thermoresponsive gels with higher crosslinking densities due to restricted chain mobility [29, 34]. It should be noted that Gel‐H_2_O/DEG exhibited non‐isotropic deswelling at temperatures higher than 40°C, with surface bubble formation due to fluid sticking to the outermost layer of the samples. This behavior has been associated with an inhomogeneous network characterized by loops and dangling chains [35, 36]. The gels also exhibited inhomogeneous color change to white, suggesting non‐uniform chain collapse at higher temperatures.

The influence of DEG content on hydrogel porosity was then investigated to better understand the observed swelling behaviors. SEM analysis of freeze‐dried samples was limited by partial collapse of the polymer network; therefore, low vacuum scanning electron microscopy (LVSEM) imaging of hydrated samples was performed. Organohydrogels did not exhibit visible porosity, likely due to the non‐swollen state, whereas equilibrium swollen hydrogel samples displayed interconnected porous structures with a web‐like polymer network (Figure 3I). This co‐continuous porosity could contribute to the high water uptake of these hydrogels. The formation of this porous morphology could be attributed to PIPS [37], as previously suggested for PNIPAM hydrogels prepared in EG [24]. Phase separation could be promoted by the partial miscibility of DEG with water and the lower affinity of PNVCL gels for DEG than for water (Figure S6A). In pure water, the equilibrium volumetric swelling ratios of Gel‐H_2_O/DEG‐50/50, Gel‐H_2_O/DEG‐40/60, Gel‐H_2_O/DEG‐30/70, and Gel‐H_2_O/DEG‐20/80 were 9, 12, 17, and 24, respectively, whereas in pure DEG, these values decreased to 8, 10, 8, and 10, respectively. These differences in swelling behavior may also partly result from the higher swelling in water observed with increasing DEG content, as discussed previously. To explore the possibility of phase separation during polymerization, UV–vis transmittance of precursor solutions with varying H_2_O/DEG ratios was monitored under intermittent UV irradiation (Figure S6B–D) [38]. The increase in transmittance for the 50/50 and 40/60 ratios likely resulted from the lower initial transmittance caused by the lower solubility of NVCL in water compared with DEG. The decrease in transmittance observed for the 30/70 and 20/80 ratios could be associated with phase separation during polymerization [38], although frozen‐in density fluctuations may also contribute [39]. The larger transmittance decrease observed for H_2_O/DEG 30/70 compared with H_2_O/DEG 20/80 (−20% and −8%, respectively) may be associated with the lower affinity of Gel‐H_2_O/DEG‐30/70 for its preparation solvent (Figure S6A) and could indicate a greater tendency for phase separation during polymerization. However, these results provided only indirect evidence for PIPS, and further investigations are required to confirm the phenomenon.

### 2.3 DLP 3D Printing of PNVCL Hydrogels

A major challenge in the DLP 3D printing of thermoresponsive hydrogels lies in balancing efficient printability with the formation of highly responsive materials [40]. Large deswelling amplitudes and fast thermoresponsive behavior generally rely on less densely crosslinked networks that facilitate water diffusion and volumetric deformation. However, reducing monomer or crosslinker concentrations to achieve such networks can compromise polymerization efficiency and mechanical integrity during printing. Herein, precursor organohydrogel solutions with H_2_O/DEG ratios of 50/50 and 20/80 (see Experimental Section and Tables 1 and 2) were used to demonstrate the DLP 3D printability of PNVCL hydrogels. DLP 3D‐printed hydrogels exhibited lower gel fractions than their UV‐cured counterparts, while additional UV post‐curing did not further increase the gel fraction (Figure S7A). This difference likely originated from the distinct irradiation conditions during DLP printing compared with bulk UV curing, including layer‐by‐layer exposure, the use of Orange G as a photoabsorber to limit light penetration and prevent overcuring beyond intended printing regions [41], and oxygen inhibition effects [42]. UV‐cured formulations containing Orange G did not form gels, whereas DLP 3D printed formulations without Orange G yielded higher gel fractions, supporting the role of the photoabsorber in reducing polymerization efficiency (Figure S7A,B). Consistent with their lower gel fractions, DLP 3D‐printed hydrogels with Orange G exhibited higher *ESR_20_* compared to UV‐cured samples (Figure S7C). The printability of the precursor solutions was further evaluated based on dimensional accuracy, print fidelity, and fabrication of complex structures. Due to the hydrophilic nature of the hydrogels, rapid swelling after printing prevented reliable measurement of the dimensions of thin samples. Therefore, dimensional accuracy was quantified using 5 × 5 × 5 mm cubic samples. The average dimensional accuracy along the X, Y, and Z axes relative to the CAD model exceeded 96% for both formulations (Figure 4A). Print fidelity and structural stability during post‐processing were assessed using hole calibration plates containing circular holes with diameters ranging from 2.5 mm to 0.25 mm (Figure 4B). All holes were fabricated and remained open after solvent exchange and swelling, and temperature‐induced contraction, indicating that the printed structure was preserved throughout these volume transitions. Finally, a gyroid was fabricated, reproducing the intended interconnected morphology and overall geometry (Figure 4C). Collectively, these results supported the compatibility of the developed precursor solution with DLP 3D printing.

**TABLE 2:** Summary of tested conditions for DLP 3D printing.

n°	Sample name	Irradiation time per layer (s)	Printed object
7^a^	Gel‐H_2_O/DEG‐50/50	150	Cube 5 × 5 × 5 mm
16	Gel‐H_2_O/DEG‐20/80	220	Cube 5 × 5 × 5 mm/hole calibration plate 7.55 × 2 mm
16	Gel‐H_2_O/DEG‐20/80	320 first layer/300	Gyroid 5 × 5 × 2 mm and 5 × 5 × 5 mm

^a^ Sample numbers correspond to those used in Table 1.

**FIGURE 4:**
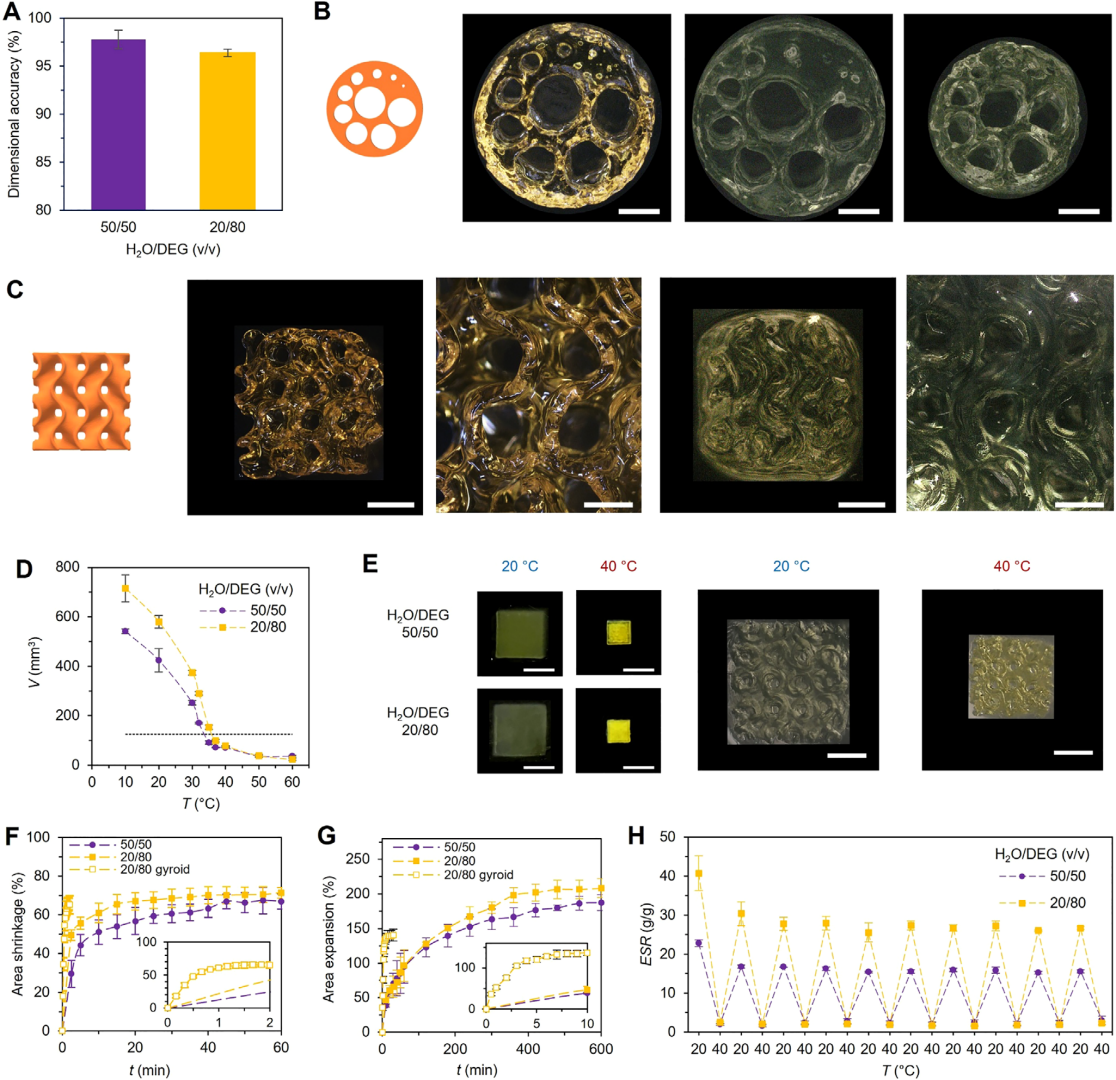
Performance of DLP 3D printed PNVCL hydrogels. (A) Dimensional accuracy of 5 × 5 × 5 mm cubic samples determined from the average dimensional error along the X, Y, and Z axes. (B) CAD model of a hole calibration plate with nominal diameters ranging from 2.5 mm to 0.25 mm, corresponding sample as‐fabricated, swollen at room temperature after solvent exchange with water, and after heating to 40°C (left to right, scale bar 2 mm). (C) CAD model of a 5 × 5 × 2 mm gyroid structure, corresponding sample as‐fabricated with magnified view, and structure after solvent exchange and swelling at room temperature with magnified view (scale bar 2 mm for overviews and 1 mm for magnified views). (D) Temperature‐induced volume change of hydrogel samples. The black dotted line indicates the volume of a 5 × 5 × 5 mm cube (125 mm^3^). (E) Area changes of cubic and gyroid samples between 20°C and 40°C (scale bar 0.5 cm for cubic samples, 1 mm for gyroid). All samples were immersed in water. (F) Time‐dependent area shrinkage during immersion in water at 40°C. (G) Time‐dependent area expansion after immersion in water at room temperature (20–23°C). (H) Cyclic deswelling and reswelling behavior at 40°C and 20°C, respectively.

The thermoresponsive behavior of DLP 3D‐printed samples was confirmed by determining temperature‐induced volume contraction, which followed the trends observed with equilibrium swelling ratio measurements (Figures 4D and S8). Deswelling and reswelling kinetics were then investigated by monitoring the area shrinkage and expansion of cubic hydrogels after sequential immersion in water baths at 40°C and 20°C, respectively (Figure 4E–G, see Experimental Section). Initial water release was faster for Gel‐H_2_O/DEG‐20/80 with area shrinkage of 50% at 2.5 min compared to 28% for Gel‐H_2_O/DEG‐50/50 at the same time, which could be attributed to the higher mesh size of Gel‐H_2_O/DEG‐20/80. However, both hydrogels reached deswelling equilibrium within 40 min and reswelling equilibrium within 6 h. Their rapid response could be attributed to the interconnected porosity, which facilitated water transport [22, 23, 24, 43]. The influence of porosity was further highlighted by DLP 3D‐printed gyroid structures, which exhibited 10‐fold and 40‐fold reductions in the time required to reach deswelling and reswelling equilibrium, respectively, compared with cubic samples (Figure 4E–G). Direct comparison of the deswelling and reswelling kinetics with those of previously reported PNVCL‐based hydrogels is challenging, as these properties are strongly influenced by hydrogel characteristics and experimental conditions, which vary across studies. In addition, some reports only provide deswelling equilibrium times, and reswelling kinetics are less frequently investigated. Table S2 summarizes the key characteristics of previously reported chemically crosslinked PNVCL hydrogels, including hydrogel system, sample dimensions, swelling ratios, response times, measurement temperatures, and mechanical properties, which are relevant for interpreting differences in thermoresponsive behavior across systems. The response times observed in this work were lower than those reported for SLA 3D‐printed PNVCL hydrogels (approximately 24 h and >40 h, respectively) [10, 11], while achieving higher *ESR_20_* and deswelling amplitude.

It should be noted that sample dimensions at reswelling equilibrium were smaller than those of the initial swollen sample size, consistent with the decrease in *ESR_20_* observed during the first deswelling/reswelling cycle (Figure 4H). This behavior can be attributed to the reduced reswelling capacity of dried hydrogels, which has been associated with irreversible collapse of the polymer network, enhanced polymer‐polymer interactions, and pore shrinkage occurring during dehydration [44, 45]. The hydrogels exhibited stable deswelling/reswelling responses from 20–40–20°C cycles 2 to 9, indicating good repeatability of the thermoresponsive behavior after the first cycle, despite their relatively low mechanical properties.

Collectively, these results demonstrated that enhanced thermoresponsive performance and improved printability were simultaneously achieved in PNVCL‐based hydrogels, addressing two major challenges in the development of these materials. Furthermore, the ability to fabricate complex 3D architectures provided opportunities for tailoring hydrogel properties.

## 3 Conclusion

This work demonstrated the preparation of PNVCL organohydrogels using DEG as a cosolvent with water, followed by solvent exchange with water, as an effective strategy for the DLP 3D printing of PNVCL hydrogels exhibiting enhanced deswelling amplitude and rapid temperature‐induced volume changes. DEG content influenced gel fraction, crosslinking density, mesh size, and swelling behavior. Further investigation of the interactions between DEG and the polymer network will provide valuable insights into the mechanisms underlying these effects. Beyond formulation optimization, the developed precursor solutions enabled the fabrication of complex DLP 3D‐printed architectures, expanding the potential of PNVCL hydrogels as thermoresponsive soft materials.

## 4 Experimental Section

### 4.1 Materials

Diethylene glycol (DEG, Sigma Aldrich), dimethylsulfoxide (DMSO, Sigma Aldrich), 3,9‐divinyl‐2,4,8,10‐tetra‐oxaspiro[5.5] undecane (DVA, ThermoFischer Scientific), ethanol (EtOH, Sigma Aldrich), N‐vinylcaprolactam (NVCL, >98.0%, Tokyo Chemical Industry), polyethylene glycol diacrylate Mn 250 (PEGDA_250_, Sigma Aldrich) and polyethylene glycol diacrylate Mn 700 (PEGDA_700_, Sigma Aldrich) were used as received without further purification. Lithium phenyl‐2,4,6‐trimethylbenzoylphosphinate (LAP) was synthesized following previously reported protocols [46]. Ultrapure water (18.2 mΩ cm at 25°C, Millipore Sigma Synergy) was used.

### 4.2 Organohydrogel Precursor Solutions

NVCL monomer, LAP photoinitiator, and PEGDA_250_, PEGDA_700_, or DVA crosslinker were added to an H_2_O/DEG water/diethylene glycol mixture and sonicated (Ultrasonic Cleaner, BPAC) until complete dissolution (∼5 min). The influence of crosslinker type, crosslinker concentration, monomer concentration, LAP concentration, and solvent composition ratio on the properties of as‐prepared hydrogels was investigated. All tested conditions are summarized in Table 1. NVCL concentration was determined relative to the volume of solvent. Crosslinker concentration was calculated relative to NVCL, and LAP concentration relative to the amount of photocrosslinkable moieties. Solvent composition ratio was defined in volume‐to‐volume ratio.

### 4.3 UV Box Preparation of Organohydrogels

The precursor solution was pipetted into Teflon molds (100 µL/mold, cylinder 10 × 1 mm) before polymerization and crosslinking under UV irradiation (Asiga Flash Cure Box, 365 nm) for 30 min.

### 4.4 Direct Light Processing (DLP) 3D Printing of Organohydrogels

Precursor solutions were used as reported in Table 1, with the addition of Orange G as a photoabsorber to achieve better resolution (2 mg mL^−1^ of precursor solution). Objects were DLP 3D printed (Asiga Max X43, KREOS, 385 nm LED) with a light irradiance of 20 mW cm^−2^ and a layer thickness of 100 µm. Irradiation time per layer was determined as the time needed for all printed objects to adhere to the printer build platform. Irradiation times per layer were dependent on the precursor solution and are summarized in Table 2. The STL models of the gyroid and the hole calibration plate were sourced from Thingiverse (Moczys and Myastremsky, respectively) under the Creative Commons Attribution‐ShareAlike (CC BY‐SA) license.

### 4.5 Gel Fraction and Swelling Tests

Gel fraction was determined using the formula *Gel fraction (%) = 100 × m_2_/m_1_*, where *m*_1_ is the initial dry mass obtained after drying the organohydrogel samples at 90°C for 2–3 days, and *m*_2_ is the dry mass after swelling the samples in excess water for over 20 h to remove unreacted components and perform solvent exchange followed by drying at 60°C overnight. Equilibrium swelling ratio at given temperature *T* (*ESR_T_*) was calculated using the formula *ESR_T_ (g/g) = (m_3,T_—m_4_)/m_4_*, where *m_3,T_* is the swollen mass after washing the organohydrogels disks in excess water for over 20 h before incubating the disks in excess water for over 20 h at given temperature *T*, and *m_4_* is the dry mass obtained after drying the swollen disks at 60°C overnight. The temperature was maintained using an oven or water bath with temperature deviations of ± 3°C. The values reported for gel fractions and *ESR* were obtained as averages over three samples.

### 4.6 Kinetics Tests

DLP 3D‐printed organohydrogels were first washed in water for over 20 h, before swelling in water at 20°C for over 20 h. To investigate hydrogel deswelling kinetics, the changes in the area of hydrogel samples were recorded over time, directly after immersing the samples in hot water (40°C, with a temperature fluctuation of ±3°C). Hydrogel reswelling kinetics were determined similarly by immersing deswollen samples in water at room temperature (20–23°C). Video data were analyzed by fitting a rectangular shape over the samples. Area shrinkage at time *t* and area expansion at time *t* were calculated using the formulae: *Area shrinkage(t) = 100 × (1 – A_t_ /A_0_)* and *Area expansion(t) = 100 × (A_t_/A_0_ – 1)*, where *A*_t_ and *A*_0_ are the areas of the hydrogel sample at time *t* and at the beginning, respectively. Reported values were obtained as averages over three samples.

### 4.7 In Situ Photorheology

Rheometer MCR 302e (Anton Paar GmbH, Graz, Austria) was equipped with a CP10 cone‐plate measurement system (diameter 10 mm). 100 µL of resin was pipetted between the plates of the device using a gap setting of 0.046 mm. The sample was oscillated at a strain of 20% and a frequency of 1 Hz at 20°C, followed by UV irradiation (500 W) after 60 s. All measurements were performed in triplicate. Gel point, defined as the crossover time between storage modulus and loss modulus curves, was reported as an average over three measurements.

### 4.8 Rheology Characterizations

Oscillatory rheological measurements were conducted using a MARS HAAKE rheometer (Thermo Fisher Scientific, Karlsruhe, Germany) on equilibrium swollen hydrogel disks (punched out 8 mm diameter) with a parallel‐plate geometry (8 mm). A normal force of ∼0.1 N was applied to ensure stable contact, and samples were maintained in a hydrated state throughout the experiment. The storage modulus *G′* was recorded within the linear viscoelastic regime at 0.1% shear strain over a frequency range of 0.1–50 Hz at 20°C. All measurements were performed in triplicate. Mesh size *ξ* (nm) and crosslinking density *n_e_* (mol cm^−3^) were estimated from the *G*′ values using the following formulae:

(1) $$G_{network}^{\prime }=\frac{G_{measured}^{\prime }}{1-\Phi_{pores}}=\frac{G_{measured}^{\prime }}{\Phi_{p}}$$

with

(2) $$\frac{1}{\Phi_{p}}=1+ESR\times \frac{\rho_{p}}{\rho_{H_{2}O}}$$

(3) $$\xi ={\left(\frac{RT}{G_{network}^{\prime }N_{A}}\right)}^{\frac{1}{3}}$$

(4) $$n_{e}=\frac{G_{network}^{\prime }}{RT}$$

where $\Phi_{pores}$ is the pore volume fraction, $\Phi_{p}$ the polymer volume fraction, ESR the equilibrium swelling ratio at 20°C, $\rho_{p}$ the density of the polymer network (1.05 g cm^−3^), $\rho_{H_{2}O}$ the (8.314 J K^−1^ mol^−1^), *T* the temperature (293.15 K). Reported values were obtained as averages over three measurements.

Low Vacuum Scanning Electron Microscopy (LVSEM) density of water (1 g cm^−3^), $N_{A}$ the Avogadro number (6.022 × 10^23^), *R* the universal gas constant

Swollen samples (3D‐printed cuboids 5 × 5 × 1.5 mm) were imaged using a FEI Quanta 100 FEG field emission scanning electron microscope (FEI, Hillsboro, USA) operated at an accelerating voltage of 10 kV under low vacuum of 0.298 torr (University of Montpellier, Platform MEA, France).

## Author Contributions

**Charlotte Dai**: investigation, writing – original draft, methodology, formal analysis. **Frédéric Demoly**: funding acquisition, validation, visualization, writing – review and editing, supervision. **Vincent Lapinte**: conceptualization, writing – review and editing, visualization, validation, supervision. **Sébastien Blanquer**: project administration, supervision, writing – review and editing, visualization, validation, conceptualization, funding acquisition.

## Conflicts of Interest

The authors declare no conflict of interest.

## Supporting information




**Supporting File**: marc70429‐sup‐0001‐SuppMat.docx.

## Data Availability

The data that support the findings of this study are available on request from the corresponding author. The data are not publicly available due to privacy or ethical restrictions.
