# Supplementary material for: Solvent‐Tuned DLP 3D‐Printed PNVCL Hydrogels With Enhanced Thermoresponse
Source: Macromol Rapid Commun. 2026 Sep 16;47(21):e70429. doi: 10.1002/marc.70429 (PMC13741390; doi:10.1002/marc.70429)
Supplement: Supplementary file 1 [file MARC-47-e70429-s001.docx]

**Supplementary Information**

**Figure S1.** (A) Solubility of NVCL monomer in various solvents. H_2_O water, DEG diethylene glycol, DMSO dimethyl sulfoxide, and EtOH ethanol. (B) ^1^H NMR analysis (400 MHz, deuterated water) of bath used to perform solvent exchange showing presence of DEG. (C) TGA curve of washed and dried hydrogel (corresponding to Gel-DVA-2 in Table 1) showing limited residual solvent (< 20%).

**Figure S2.** (A) ^1^H NMR analysis (400 MHz, deuterated water) of bath used to wash the organohydrogels showing presence of polymer and residual monomer. (B) Influence of PEGDA_700_ crosslinker concentration on *ESR_20_* and *ESR_40_*. (C) Corresponding gel diameter changes. See Experimental Section and Table 1 for gel preparation conditions.

**Figure S3. Effect of monomer concentration on properties of PNVCL hydrogels. (**A) Gel fraction. (B) *ESR_20_* and *ESR_40_*. (C) Corresponding gel diameter changes. See Experimental Section and Table 1 for gel preparation conditions.

**Figure S4.** (A) Evolution of LAP absorbance with increasing DEG content. LAP concentration was 1 mg mL^-1^ to avoid optical saturation. (B) Influence of LAP concentration on gel fraction. See Experimental Section and Table 1 for gel preparation conditions.

| LAP (w/w%) | 0.5 | 2 | 4 |
| --- | --- | --- | --- |
| Gel point (s) | 60 ± 0 | 56.0 ± 0.1 | 65.5 ± 0.9 |
| *G’_plateau_* (kPa) | 9.3 ± 1.0 | 15.6 ± 1.0 | 8.2 ± 0.2 |

**Table S1**. Influence of LAP concentration on gel point and plateau storage modulus. See Experimental Section and Table 1 for precursor solution compositions.

**Figure S5.** DSC thermograms of Gel-H_2_O/DEG. Dried hydrogels were weighed into hermetic pans, followed by the addition of water at a 1:1 hydrogel:water mass ratio. After cooling to -10°C, temperature was increased to 60 °C at a rate of 1 K min^-1^ for three cycles. Reported data correspond to the second heating cycle.

**Figure S6.** (A) Volumetric swelling ratio *Q_v_* of Gel-H_2_O/DEG in H_2_O/DEG mixtures. *Q_v_* (V_solvent_/V_polymer_) was determined from washed and dried hydrogels samples after over 48 h swelling to ensure equilibrium. Polymer density was estimated to 1.05 g cm^3^. Photographs correspond to Gel-H_2_O/DEG-50/50. (B-D) Influence of initial solvent composition ratio on relative transmittance change as a function of time for non-irradiated precursor solutions (B) and intermittently irradiated solutions (C), with (D) showing a magnified view of (C). The onset of polymerization was used as the reference point. Relative change in transmittance was calculated as the normalized difference between the transmittance at time t, and the transmittance of the non-irradiated sample. Relative changes in transmittance were calculated at 500 nm and averaged over three measurements. See Experimental Section and Table 1 for precursor solution compositions.

**Figure S7.** (A) Gel fraction of hydrogels prepared by UV curing and DLP 3D printing. Orange G+ and Orange G- correspond to precursor solutions with and without the photoabsorber, respectively. (B) Photographs of precursor solutions before UV curing (top) and the corresponding hydrogels after UV curing (bottom). H_2_O/DEG 50/50 Orange G-, H_2_O/DEG 20/80 Orange G-, H_2_O/DEG 50/50 Orange G+, and H_2_O/DEG 20/80 Orange G+ (left to right). (C) *ESR_20_* and *ESR_40_* of hydrogels prepared by UV curing and DLP 3D printing. See the Experimental Section and Table 1 for precursor solution compositions.

**Figure S8.** Temperature-induced change in *ESR* of DLP 3D-printed hydrogels.

**Table S2. Summary of properties of chemically crosslinked PNVCL-based hydrogels.** Abbreviations: AM, acrylamide; BIS, N,N′-methylenebisacrylamide; CMC, carboxymethyl cellulose; DEG diethylene glycol; DEGDA, diethylene glycol diacrylate; DMAM, dimethylacrylamide; DVA, 3,9-divinyl-2,4,8,10-tetraoxaspiro[5.5]undecane; DVI, 1,3-divinylimidazolidin-2-one; EGDMA, ethylene glycol dimethacrylate; HEMA, 2-hydroxyethyl methacrylate; H-ESO-HEC, hydrolyzed epoxy soybean oil-grafted hydroxyethyl cellulose; H₂O, water; NIPAM, N-isopropylacrylamide; NVP, N-vinylpyrrolidone; SLA stereolithography, VAC, vinyl acetate.

a. Equilibrium swelling ratio in swollen and shrunken state, with temperatures provided in parentheses. Values were calculated as *ESR = (m_swollen_ - m_dry_)/m_dry_*. Values marked with V were determined from volume ratio.

b. Time to reach deswelling equilibrium (first line) and reswelling equilibrium (second line). Values were determined from mass swelling measurements, except for values marked with A which were determined from projected surface area changes. < x: authors reported equilibrium was reached in less than x time. x (reported eq.): authors stated equilibrium after x time. ≤ x: estimated from cyclic temperature-switching measurements, no dedicated kinetic measurements were reported.

c. Values correspond to the storage modulus *G′*, except for values marked with *E* which correspond to Young modulus.

Reported values were rounded to the nearest unit for consistency. Reference numbers correspond to the citations in the main text.

| System | Solvent | Sample characteristics | ESR^a^ (g/g) | Equilibrium time^b^ | Mechanical property (Pa)^c^ | Refs. |
| --- | --- | --- | --- | --- | --- | --- |
| H-ESO-HEC-P(NVCL-DMAM-BIS) | Aqueous emulsion | Cylindrical  - | 22 - 10  (20 - 60 °C) | ~150 min (20 - 60 °C)  - | - | [13] |
| CMC-P(NVCL-AM-BIS) | Aqueous emulsion | - | 16 - 8  (20 - 60 °C) | ~200 min (25 - 60 °C)  - | ~10^3^ | [14] |
| SLA P(NVCL-VAC-DEGDA) | No solvent | Cylindrical  40 x 5 mm | 2 - 1  (20 - 50 °C) | 24 h (reported eq., 22 - 50 °C)  >40 h (50 - 22 °C) | - | [10] |
| SLA P(NVCL-DMAM-EGDMA) | No solvent | Jigsaw  45 x 45 x 2 mm | 4 - 2  (20 - 50 °C) | 24 h (reported eq., 20 - 50 °C)  24 h (reported eq., 50 - 20 °C) | 2 x 10^6 E^ | [11] |
| P(NVCL-NIPAM-EGDMA)-clay | No solvent | Cylindrical  30 x 4 mm | 2 - 1  (20 - 50 °C) | ≤24 h (20 - 50 °C)  ≤24 h (20- 50 °C) | 2 x 10^5^ | [18] |
| P(NVCL-NIPAM-EGDMA) | No solvent | Cylindrical  30 x 4 mm | 2 - 1  (20 - 50 °C) | <24 h (20 - 50 °C)  24h (reported eq., 20 - 50 °C) | 2 x 10^5^ | [19] |
| P(NVCL-NVP-EGDMA) | No solvent | Cylindrical  - | 7 - 4  (20 - 50 °C) | ≤24 h (20 - 50 °C)  ≤24 h (20 - 50 °C) | - | [20] |
| P(NVCL-DEGDA) | No solvent | Cylindrical  30 x 4 mm | 3 - 1  (20 - 50 °C) | ~160 h (20 - 50 °C)  ~30 h (50 - 20 °C) | 5 x 10^8 E^ | [21] |
| P(NVCL-BIS) | EtOH/H_2_O | - | 30 - 3^SR^  (20 - 40 °C) | - | - | [15] |
| P(NVCL-DVA) | EtOH/H_2_O | Cylindrical  2 mm thick | 4 - 1^V^  (20 - 37 °C) | - | 2 x 10^3 E^ | [16] |
| P(NVCL-DVI-EGDMA) | EtOH | Cylindrical  4 mm thick | 6 - 2  (20 - 37 °C) | - | - | [12] |
| P(NVCL-HEMA-DVI-EGDMA) | EtOH | Cylindrical  0.3 mm thick | 8 - 1  (20 - 40 °C) | - | - | [17] |
| P(NVCL-DVA) | H_2_O/DEG | Cylindrical  10 x 1 mm | 13 - 1  (20 - 40 °C) | - | ~10^3^ | This work  (Gel-DVA-2) |
| DLP P(NVCL-DVA) | H_2_O/DEG | Cube  5 x 5 x 5 mm | 22 - 2  (20 - 40 °C) | 40 min (20 - 40 °C)^A^  6 h (40 - 20 °C)^A^ | - | This work (Gel-H_2_O/DEG-50/50) |
| DLP P(NVCL-DVA) | H_2_O/DEG | Cube  5 x 5 x 5 mm | 36 - 2  (20 - 40 °C) | 40 min (20 - 40 °C)^A^  6 h (40 - 20 °C)^A^ | - | This work (Gel-H_2_O/DEG-20/80) |
